# Supplementary material for: Site climate more than soil properties and topography shape the natural arbuscular mycorrhizal symbiosis in maize and spore density within rainfed maize (Zea mays L.) cropland in the eastern DR Congo
Source: PLoS One. 2024 Dec 13;19(12):e0312581. doi: 10.1371/journal.pone.0312581 (PMC11642996; doi:10.1371/journal.pone.0312581)
Supplement: S1 Table — (DOCX) [file pone.0312581.s001.docx]

**S1 Table.** Descriptive statistics of climatic parameters, physical and chemical soil properties, and topographical and vegetation parameters used, alongside mycorrhization parameters

| **Paramètres (unites)** | **Kabare** | **Uvira** | **Walungu** | **Moyenne** | ***P value*** |
| --- | --- | --- | --- | --- | --- |
| Altitude (m) | 1818 ± 173 | 859 ± 52 | 1750 ± 349 | 1471 ± 491 | *<0,001**** |
| T° max (°C) | 23,09 ± 0,78 | 29,83 ± 0,41 | 24,6 ± 1,93 | 25,78 ± 3,2 | *<0,001**** |
| T° min (°C) | 12,22 ± 0,51 | 18,15 ± 0,79 | 13,55 ± 1,46 | 14,59 ± 2,79 | *<0,001**** |
| T° mean (°C) | 17,63 ± 0,63 | 23,93 ± 0,55 | 19,04 ± 1,69 | 20,15 ± 2,97 | *<0,001**** |
| SRadiation (kJ m^-2^ jour^-1^) | 14858 ± 295 | 15975 ± 71 | 15274 ± 300 | 15346 ± 543 | *<0,001**** |
| Vapor P (Kpa) | 1,42 ± 0,14 | 1,75 ± 0,02 | 1,48 ± 0,18 | 1,55 ± 0,2 | *<0,001**** |
| Wind speedt (m/s) | 1,85 ± 0,13 | 2,13 ± 0,04 | 2,03 ± 0,05 | 1,99 ± 0,15 | *<0,001**** |
| Rainfall (mm) | 1675 ± 117 | 955 ± 61 | 1536 ± 203 | 1393 ± 348 | *<0,001**** |
| NDVI | 0,42 ± 0,06 | 0,42 ± 0,06 | 0,4 ± 0,08 | 0,41 ± 0,07 | *0,496 ns* |
| Soil Ph | 5,44 ± 0,55 | 5,85 ± 0,5 | 5,23 ± 0,46 | 5,53 ± 0,57 | *<0,001**** |
| CEC | 25,15 ± 6,6 | 19 ± 4,25 | 24,29 ± 7,1 | 22,82 ± 6,6 | *<0,001**** |
| Soil density (g.cm^-3^) | 0,9 ± 0,51 | 0,59 ± 0,36 | 1 ± 0,52 | 0,82 ± 0,49 | *0,003 *** |
| WC | 27,56 ± 3,64 | 22,45 ± 4,15 | 27,88 ± 3,92 | 25,89 ± 4,59 | *<0,001**** |
| K | 1,38 ± 0,44 | 0,97 ± 0,58 | 1,65 ± 0,42 | 1,31 ± 0,55 | *<0,001**** |
| Ca | 4,93 ± 2,62 | 6,53 ± 3,11 | 3,59 ± 1,79 | 5,15 ± 2,84 | *<0,001**** |
| Mg | 2,6 ± 1,76 | 3,52 ± 1,68 | 1,95 ± 0,83 | 2,76 ± 1,66 | *0,001 *** |
| Na | 0,82 ± 1,38 | 1,63 ± 1,55 | 0,29 ± 0,55 | 0,97 ± 1,39 | *0,001 *** |
| N (%) | 0,56 ± 0,32 | 0,62 ± 0,27 | 0,46 ± 0,26 | 0,55 ± 0,29 | *0,109 ns* |
| TSB | 9,92 ± 6,84 | 12,36 ± 6,76 | 6,71 ± 2,91 | 9,96 ± 6,39 | *0,003 *** |
| Clay (%) | 40,82 ± 7,56 | 30,7 ± 7,92 | 41,04 ± 7,68 | 37,4 ± 9,06 | *<0,001**** |
| Sand (%) | 36,51 ± 8,39 | 48,39 ± 9 | 37,25 ± 9,55 | 40,78 ± 10,4 | *<0,001**** |
| Silt (%) | 22,67 ± 3,44 | 20,91 ± 3,61 | 21,71 ± 2,8 | 21,82 ± 3,41 | *0,090 ns* |
| Freq (%) | 52.1± 10.37 | 26.40± 7.26 | 44.7± 11,03 | 41,08 ± 12,66 | *0,016 *** |
| Intens (%) | 12.6± 0,8 | 6.22± 0,27 | 13.30± 0,8 | 10,7 ± 0,71 | *0,002 *** |
| AM spores density | 165±26.1 | 329±79 | 156±48 | 216.7 ± 7,18 | *<0,001**** |

The results revealed significant variations in climatic parameters among territories. Specifically, with the exception of vapour pressure, where no difference was observed between Kabare and Walungu, all climatic parameters exhibited significant variability among territories (p<0.05). These parameters include maximum temperature (p<0.0001), minimum temperature (p<0.0001), average temperature (p<0.0001), wind speed (p<0.0001), precipitation (p<0.0001), and solar radiation (p<0.0001, **Figure 5A, B**). The Uvira territory, situated at a lower altitude, is characterized by elevated values of temperature, wind speed, vapour pressure, and solar radiation, sequentially followed by Walungu and Kabare. In contrast, Uvira experiences lower precipitation (⁓900mm) compared to Kabare and Walungu. Kabare (high and medium altitude) demonstrated high precipitation (⁓1800mm), succeeded by Walungu (high and medium altitude) and Uvira (low altitude). Similarly, a significant differentiation among territories was observed based on soil physical properties, specifically clay content (p<0.0001), sand content (p<0.0001), silt content (p<0.004), soil water content (p<0.0001), soil density (p<0.0001), and field capacity (p<0.0001, **Figure 6A**). Indeed, the Kabare territory exhibited high values for soil physical properties, followed by Walungu and Uvira, except for sand content, which is higher in Walungu and Uvira.

Concerning soil chemical properties, only five parameters facilitated complete discrimination among territories, including CEC (p<0.0001), soil pH (p<0.0001), carbon content (p<0.0001), organic matter content (p<0.0001), and the C/N ratio (p<0.0001). Specifically, soil pH and CEC are higher in Kabare, followed by Uvira and Walungu, while carbon content, organic matter content, and the C/N ratio are higher in Uvira, followed by Kabare and Walungu. However, limited discrimination among territories was observed for other soil chemical parameters such as base saturation rate, potassium content, calcium content, magnesium content, and sodium content, showing similarity between the Kabare and Uvira territories with low values compared to Walungu, except for potassium content (**Figure 6B**). The soil phosphorus content was notably high in Uvira compared to other territories; however, no significant difference was observed between the Kabare and Walungu territories for soil phosphorus content
