# Supplementary material for: Site climate more than soil properties and topography shape the natural arbuscular mycorrhizal symbiosis in maize and spore density within rainfed maize (Zea mays L.) cropland in the eastern DR Congo
Source: PLoS One. 2024 Dec 13;19(12):e0312581. doi: 10.1371/journal.pone.0312581 (PMC11642996; doi:10.1371/journal.pone.0312581)
Supplement: S2 Table — (DOCX) [file pone.0312581.s002.docx]

S2 Table. Pearson correlation matrix (correlation coefficient R) illustrating the relationships between climatic features, physical and chemical soil properties, topographic parameters, mycorrhizal colonization of maize, as well as the density of AMF

| **Variable** | **CEC** | **Fieldcap** | **Clay** | **Slope** | **Intens** | **Freq** | **Alt** | **Rainfall** | **TSB** | **ON** | **pH** | **Sand** | **wind** | **Srad** | **Tmin** | **Tmax** | **Tmean** | **P** | **C** | **MO** | **CTI** | **Sp_dens** | **NDVI** | **C.N** |
| --- | --- | --- | --- | --- | --- | --- | --- | --- | --- | --- | --- | --- | --- | --- | --- | --- | --- | --- | --- | --- | --- | --- | --- | --- |
| **CEC** | 1 |  |  |  |  |  |  |  |  |  |  |  |  |  |  |  |  |  |  |  |  |  |  |  |
| **Fieldcap** | 0.72 | 1 |  |  |  |  |  |  |  |  |  |  |  |  |  |  |  |  |  |  |  |  |  |  |
| **Clay** | 0.71 | 0.75 | 1 |  |  |  |  |  |  |  |  |  |  |  |  |  |  |  |  |  |  |  |  |  |
| **Slope** | 0.17 | 0.039 | 0.13 | 1 |  |  |  |  |  |  |  |  |  |  |  |  |  |  |  |  |  |  |  |  |
| **Intens** | 0.035 | -0.047 | 0.088 | 0.3 | 1 |  |  |  |  |  |  |  |  |  |  |  |  |  |  |  |  |  |  |  |
| **Freq** | 0.27 | 0.18 | 0.24 | 0.37 | **0.76** | 1 |  |  |  |  |  |  |  |  |  |  |  |  |  |  |  |  |  |  |
| **Alt** | 0.27 | 0.12 | 0.12 | 0.53 | **0.77** | **0.78** | 1 |  |  |  |  |  |  |  |  |  |  |  |  |  |  |  |  |  |
| **Rainfall** | 0.36 | 0.21 | 0.17 | 0.52 | **0.7** | **0.79** | 0.96 | 1 |  |  |  |  |  |  |  |  |  |  |  |  |  |  |  |  |
| **TSB** | -0.34 | -0.48 | -0.57 | 0.064 | 0.19 | 0.026 | 0.12 | 0.054 | 1 |  |  |  |  |  |  |  |  |  |  |  |  |  |  |  |
| **ON** | -0.23 | -0.36 | -0.47 | 0.0088 | 0.13 | 0.017 | 0.15 | 0.072 | 0.83 | 1 |  |  |  |  |  |  |  |  |  |  |  |  |  |  |
| **pH** | -0.6 | -0.76 | -0.78 | -0.086 | 0.083 | -0.17 | -0.096 | -0.17 | 0.8 | 0.63 | 1 |  |  |  |  |  |  |  |  |  |  |  |  |  |
| **Sand** | -0.74 | -0.8 | -0.95 | -0.12 | -0.091 | -0.27 | -0.15 | -0.2 | 0.41 | 0.36 | 0.7 | 1 |  |  |  |  |  |  |  |  |  |  |  |  |
| **wind** | -0.5 | -0.43 | -0.38 | -0.36 | **-0.5** | **-0.64** | -0.72 | -0.83 | 0.13 | 0.13 | 0.39 | 0.45 | 1 |  |  |  |  |  |  |  |  |  |  |  |
| **Sradiation** | -0.43 | -0.31 | -0.28 | -0.48 | **-0.64** | **-0.76** | -0.9 | -0.97 | 0.056 | 0.025 | 0.28 | 0.32 | 0.92 | 1 |  |  |  |  |  |  |  |  |  |  |
| **Tmin** | -0.39 | -0.19 | -0.19 | -0.52 | **-0.69** | **-0.79** | -0.94 | -0.98 | -0.045 | -0.078 | 0.16 | 0.21 | 0.79 | 0.95 | 1 |  |  |  |  |  |  |  |  |  |
| **Tmax** | -0.38 | -0.2 | -0.2 | -0.51 | **-0.7** | **-0.78** | -0.94 | -0.98 | -0.024 | -0.061 | 0.17 | 0.22 | 0.79 | 0.95 | 0.99 | 1 |  |  |  |  |  |  |  |  |
| **Tmean** | -0.39 | -0.2 | -0.2 | -0.52 | **-0.69** | **-0.79** | -0.94 | -0.99 | -0.034 | -0.069 | 0.17 | 0.22 | 0.79 | 0.95 | 1 | 1 | 1 |  |  |  |  |  |  |  |
| **P** | -0.23 | -0.19 | -0.16 | -0.33 | **-0.39** | **-0.51** | -0.57 | -0.57 | 0.3 | 0.2 | 0.33 | 0.14 | 0.5 | 0.61 | 0.55 | 0.58 | 0.57 | 1 |  |  |  |  |  |  |
| **C** | -0.34 | -0.31 | -0.29 | -0.17 | **-0.58** | **-0.67** | -0.56 | -0.58 | 0.12 | 0.05 | 0.31 | 0.3 | 0.52 | 0.58 | 0.56 | 0.58 | 0.57 | 0.56 | 1 |  |  |  |  |  |
| **MO** | -0.34 | -0.31 | -0.29 | -0.17 | **-0.58** | **-0.67** | -0.56 | -0.58 | 0.12 | 0.05 | 0.31 | 0.3 | 0.52 | 0.58 | 0.56 | 0.58 | 0.57 | 0.56 | 1 | 1 |  |  |  |  |
| **CTI** | -0.27 | -0.22 | -0.3 | -0.5 | -0.32 | -0.29 | -0.43 | -0.42 | 0.065 | -0.0046 | 0.23 | 0.29 | 0.35 | 0.43 | 0.41 | 0.42 | 0.42 | 0.26 | 0.27 | 0.27 | 1 |  |  |  |
| **Sp_dens** | -0.27 | -0.29 | -0.23 | -0.3 | **-0.42** | **-0.39** | **-0.56** | **-0.5** | 0.18 | 0.038 | 0.28 | 0.12 | 0.28 | **0.45** | **0.47** | **0.5** | **0.48** | **0.39** | 0.34 | 0.34 | **0.41** | 1 |  |  |
| **NDVI** | 0.13 | 0.0012 | -0.057 | -0.2 | -0.27 | -0.18 | -0.18 | -0.079 | -0.16 | -0.23 | -0.048 | 0.086 | 0.0045 | 0.046 | 0.066 | 0.048 | 0.056 | -0.011 | 0.029 | 0.029 | 0.22 | 0.16 | 1 |  |
| **C.N** | -0.021 | 0.18 | 0.22 | -0.046 | **-0.41** | **-0.39** | -0.41 | -0.38 | -0.52 | -0.63 | -0.36 | -0.12 | 0.22 | 0.31 | 0.38 | 0.38 | 0.38 | 0.21 | 0.55 | 0.55 | 0.066 | 0.044 | 0.11 | 1 |
