# Supplementary material for: Site climate more than soil properties and topography shape the natural arbuscular mycorrhizal symbiosis in maize and spore density within rainfed maize (Zea mays L.) cropland in the eastern DR Congo
Source: PLoS One. 2024 Dec 13;19(12):e0312581. doi: 10.1371/journal.pone.0312581 (PMC11642996; doi:10.1371/journal.pone.0312581)
Supplement: S3 Table — (DOCX) [file pone.0312581.s003.docx]

| **Latent variable** | **Predictors variables** | **Weight** | **Sd of**  **weight** | ***p*-value Weight** | **Loading** | **Sd of Loading** | ***p*-value of Loading** |
| --- | --- | --- | --- | --- | --- | --- | --- |
| Climate | Elevation/Altitude | **-0.996** | 0.3 | 0.001 | **-0.98** | 0.013 | <0.0001 |
|  | Rainfall | -0.69 | 0.7 | 0.41 | -0.95 | 0.03 | <0.0001 |
|  | Tmean | 13.7 | 19.3 | 0.5 | 0.95 | 0.003 | <0.0001 |
|  | Tmax | -7.1 | 10.3 | 0.51 | 0.94 | 0.003 | <0.0001 |
|  | Tmin | -5.9 | 9.1 | 0.54 | 0;94 | 0.003 | <0.0001 |
|  | Sradiation | -0.23 | 0.77 | 0.84 | 0.89 | 0.046 | <0.0001 |
|  | Wind | 0.22 | 0.33 | 0.61 | 0.74 | 0.07 | <0.0001 |
| Soil physical properties | FieldCap | -0.12 | 0.9 | 0.3 | 0.34 | 0.23 | 0.17 |
|  | Sand | -033 | 1.9 | 0.34 | -0.48 | 0.59 | 014 |
|  | Clay content | 0.25 | 1.3 | 0.8 | 0.47 | 0.51 | 0.13 |
| Soil chemical properties | C | **0.84** | 0.17 | <0.0001 | 0.89 | 0.06 | <0.0001 |
|  | C/N | -0.18 | 0.25 | 0.44 | 0.57 | 0.1 | <0.0001 |
|  | CEC | -0.06 | 0.14 | 0.7 | -0.26 | 0.13 | 0.04 |
|  | N | 0.13 | 0.22 | 0.53 | -0.09 | 0.15 | 0.58 |
|  | P | **-0.34** | 0.13 | 0.007 | 0.65 | 0.1 | <0.0001 |
|  | TSB | **-0.53** | 0.25 | 0.02 | -0.13 | 0.16 | 0.43 |
|  | pH | -0.02 | 0.29 | 0.96 | 0.10 | 0.15 | 0.46 |
| Topography and vegetation | NDVI | 0.31 | 0.18 | 0.07 | 0.49 | 0.23 | 0.02 |
|  | CTI | 0.36 | 0.24 | 0.1 | 0.68 | 0.33 | 0.02 |
|  | Slope | -0.49 | 0.38 | 0.11 | -0.74 | 0.42 | 0.04 |
| Path model for AMF root colonization | | Path coefficient | | Sd of Path  coefficient | T-values | *p*-values | R^2^ value |
| Site climate | AMF root colonization | -0.63 | | 0.078 | 8.07 | <0.0001 | 0.77 |
| Soil Physical properties | AMF root colonization | 0.1 | | 0.04 | 1 | 0.32 |  |
| Soil chemical properties | AMF root colonization | -0.31 | | 0.08 | 4.09 | <0.0001 |  |
| Topography and Vegetation | AMF root colonization | 0.024 | | 0.07 | 0.59 | 0.55 |  |

S3 Table. PLS-SEM model results for the effect of predictor variable on maize roots colonization showing the weight, loading values, and the path coefficients of the model and their associated p-values
