# Supplementary material for: Site climate more than soil properties and topography shape the natural arbuscular mycorrhizal symbiosis in maize and spore density within rainfed maize (Zea mays L.) cropland in the eastern DR Congo
Source: PLoS One. 2024 Dec 13;19(12):e0312581. doi: 10.1371/journal.pone.0312581 (PMC11642996; doi:10.1371/journal.pone.0312581)
Supplement: S5 Table — (PDF) [file pone.0312581.s005.pdf]

Id	Terr	Villages	Latitude	Longitude	Altitude (m)	Tmax °C
1	Kabare	Bugobe cifuma	-2.510584	28.764086	1967.776855	22.375
2	Kabare	Bugobe cifuma	-2.50901	28.758858	1968.909424	22.2166996
3	Kabare	Bugobe cifuma	-2.510104	28.762519	1974.998291	22.4500008
4	Kabare	Bugobe kahave	-2.506785	28.757327	1975.724121	22.2166996
5	Kabare	Bugobe kahave	-2.50444	28.757252	1982.464355	22.2166996
6	Kabare	Bugobe kahave	-2.501986	28.7597	1935.210205	22.4249992
7	Kabare	Bushumba	-2.35437	28.807955	1608.7	24.1166992
8	Kabare	Bushumba	-2.324187	28.816378	1699.4	23.9249992
9	Kabare	Bushumba	-2.354187	28.806378	1701.4	24.1166992
10	Kabare	Bushwira Cit	-2.43858	28.762918	2024.512207	21.8416996
11	Kabare	Bushwira Cit	-2.443985	28.779479	1953.838379	22.8332996
12	Kabare	Bushwira Cit	-2.438519	28.762144	2039.35498	21.8416996
13	Kabare	Bushwira CE	-2.439086	28.769651	2011.466309	22.5249996
14	Kabare	Bushwira CE	-2.439165	28.768982	2018.743164	22.5249996
15	Kabare	Bushwira CE	-2.459165	28.809818	2019.463164	24.3167
16	Kabare	Cirunga Kar	-2.503475	28.781185	1974.171143	22.4333
17	Kabare	Cirunga Kar	-2.503847	28.780724	1982.131836	22.4333
18	Kabare	Cirunga Kar	-2.503835	28.78378	1981.911133	22.4333
19	Kabare	Cirunga Mul	-2.487693	28.784641	1945.171875	22.6749992
20	Kabare	Cirunga Mul	-2.489527	28.784154	1951.334229	22.6749992
21	Kabare	Cirunga Mul	-2.492329	28.782536	1985.865723	22.6749992
22	Kabare	Katana	-2.22341	28.84583	1623.408203	23.3332996
23	Kabare	Katana	-2.22341	28.84583	1548.5	23.3332996
24	Kabare	Katana	-2.222793	28.847053	1516.4	23.3332996
25	Kabare	Kavumu	-2.237712	28.89582	1772.5	23.0417004
26	Kabare	Kavumu	-2.335632	28.788992	1863.5	23.7583008
27	Kabare	Kavumu	-2.296712	28.80382	1732.5	23.6583004
28	Kabare	Tchibati	-2.243773	28.818713	1652.3	23.5249996
29	Kabare	Tchibati	-2.238332	28.803752	1754.8	23.1583004
30	Kabare	Tchibati	-2.283167	28.766052	1754.8	20.2082996
31	Kabare	Miti	-2.3363	28.768687	1902.5	21.8250008
32	Kabare	Miti	-2.325953	28.811652	1682.6	23.9249992
33	Kabare	Miti	-2.35175	28.782813	1771.9	23.5832996
34	Kabare	Mudaka	-2.400312	28.815713	1555.4	24.6166992
35	Kabare	Mudaka	-2.400098	28.817177	1568	24.6166992
36	Kabare	Mudaka	-2.335632	28.788992	1694	23.7583008
37	Kabare	Luhihi	-2.283863	28.887222	1543.8	23.5583
38	Kabare	Luhihi	-2.284189	28.907222	1620.3	23.3917007
39	Kabare	Luhihi	-2.283989	28.879222	1660.2	23.5832996
40	Walungu	Burhale	-2.709488	28.639184	1841.1	25.0583
41	Walungu	Burhale	-2.71049	28.633933	1987.695557	25.1333008
42	Walungu	Burhale	-2.7107	28.632631	1991.552246	25.1333008
43	Walungu	Butuza	-2.701494	28.658623	1986.468262	24.9083004
44	Walungu	Butuza	-2.689891	28.660374	1975.414551	24.8167
45	Walungu	Butuza	-2.707374	28.656767	1902.5	25.1082993
46	Walungu	Kamanyola	-2.766137	28.999695	900.4	29.0167007
47	Walungu	Kamanyola	-2.753128	29.001385	912.8	28.8917007
48	Walungu	Kamanyola	-2.76572	29.000087	898.9	29.0167007
49	Walungu	Luciga	-2.717947	28.767403	1568	23.1000004

50	Walungu	Luciga	-2.697947	28.707403	1841.1	24.0667
51	Walungu	Luciga	-2.697947	28.696403	1795.1	24
52	Walungu	Lurhala	-2.620543	28.74572	1967	22.5417004
53	Walungu	Lurhala	-2.622609	28.750745	1968.58	22.5417004
54	Walungu	Lurhala	-2.622378	28.750409	1997.5	22.5417004
55	Walungu	Mugogo	-2.5978	28.755233	1997.5	22.4417
56	Walungu	Mugogo	-2.601	28.6568	1989.5	23.1166992
57	Walungu	Mugogo	-2.578	28.715687	1992.5	23.2000008
58	Walungu	WC	-2.60626	28.65802	1836.5	23.3167
59	Walungu	WC	-2.623682	28.668972	1768.1	23.7166996
60	Walungu	WC	-2.616047	28.68665	1737.9	24.0832996
61	Walungu	Mushinga	-2.75222	28.66585	1801.6	25.0417004
62	Walungu	Mushinga	-2.73622	28.69385	1601.8	24.8083
63	Walungu	Mushinga	-2.74322	28.67185	1742.4	24.8332996
64	Uvira	Bwegera	-2.91552	29.057802	951.5	29.5667
65	Uvira	Bwegera	-2.913685	29.0588	924.5	29.5667
66	Uvira	Bwegera	-2.889365	29.046758	924.7	29.6667004
67	Uvira	Kabunambo	-3.065507	29.236303	812.6	30.0167007
68	Uvira	Kabunambo	-3.130442	29.433267	832.6	30.5499992
69	Uvira	Kabunambo	-3.065507	29.366666	868.6	30.1583004
70	Uvira	Katogota	-2.796165	28.999672	902.5	29.1417007
71	Uvira	Katogota	-2.775518	28.999178	889.7	28.9249992
72	Uvira	Katogota	-2.695183	28.890133	919.8	24.5499992
73	Uvira	Kigurwe	-3.061883	29.201485	862	30.0417004
74	Uvira	Kigurwe	-3.088333	29.21485	856	30.3917007
75	Uvira	Kigurwe	-3.046188	29.173485	850	30.0249996
76	Uvira	Kiliba	-3.328482	29.215012	745.1	29.6499996
77	Uvira	Kiliba	-3.319195	29.213218	779.8	29.7833004
78	Uvira	Kiliba	-3.317267	29.212307	754.1	29.9167004
79	Uvira	Lubarika	-2.822162	28.968918	907.1	29.0499992
80	Uvira	Lubarika	-2.823488	28.96323	913.3	29.0499992
81	Uvira	Lubarika	-2.837457	29.002428	888.6	29.3999996
82	Uvira	Luvungi	-2.843083	29.013312	882.5	29.4333
83	Uvira	Luvungi	-2.859117	29.029968	890.4	29.5750008
84	Uvira	Luvungi	-2.859215	29.029987	875	29.5750008
85	Uvira	Ndunda	-3.066955	29.239085	810.1	30.0167007
86	Uvira	Ndunda	-3.068493	29.240775	809.7	30.0167007
87	Uvira	Ndunda	-3.067782	29.242918	803.8	30.0167007
88	Uvira	Rusabagi	-3.053623	29.228055	827.2	30.0167007
89	Uvira	Rusabagi	-3.056583	29.228627	816.7	30.0167007
90	Uvira	Rusabagi	-3.058403	29.22812	817	30.0167007
91	Uvira	Sange	-3.065248	29.241995	805.4	30.0167007
92	Uvira	Sange	-3.04393	29.189502	854.4	30.0499992
93	Uvira	Sange	-3.16393	29.189502	876.4	30.5249996
94	Uvira	Sasira	-3.047518	29.213262	918.7	29.9750004
95	Uvira	Sasira	-3.107667	29.335163	921.8	30.3416996
96	Uvira	Sasira	-3.047518	29.132612	925.9	29.8332996

Tmean °C	wind (m/s)	VaporP (kPa)	Pmm	liation (kJ m-2 da	Tmin °C	CTI
17.1000004	1.7181799	1.3200001	1772	14597	11.8500004	6.7179399
16.9666996	1.7181799	1.3200001	1782	14574	11.7250004	5.37572
17.1417007	1.7181799	1.3200001	1761	14635	11.8583002	6.7992001
16.9666996	1.72727	1.3200001	1782	14574	11.7250004	7.2010002
16.9666996	1.72727	1.3200001	1782	14574	11.7250004	5.2825699
17.1082993	1.70909	1.33	1767	14634	11.8500004	5.51334
18.3999996	1.9454499	1.46	1555	15153	12.7666998	5.7893
18.2000008	1.80909	1.42	1679	14879	12.5500002	6.36515
18.3999996	1.9454499	1.46	1555	15153	12.7666998	5.7878399
16.5249996	1.72727	1.3	1819	14422	11.2749996	5.9987402
17.3500004	1.8	1.34	1759	14649	11.9417	5.4734101
16.5249996	1.72727	1.3	1819	14422	11.2749996	5.5164399
17.0667	1.7	1.3200001	1792	14525	11.6750002	5.72229
17.0667	1.7	1.3200001	1792	14525	11.6750002	5.8199801
18.7583008	2.0272701	1.42	1547	15281	13.1999998	6.7254901
17.125	1.86364	1.3200001	1733	14730	11.8500004	6.2894001
17.125	1.86364	1.3200001	1733	14730	11.8500004	6.1603699
17.125	1.86364	1.3099999	1733	14730	11.8500004	8.29877
17.3250008	1.89091	1.34	1718	14779	11.9833002	6.7055898
17.3250008	1.89091	1.34	1718	14779	11.9833002	5.8632998
17.3250008	1.86364	1.3200001	1718	14779	11.9833002	6.7569199
17.75	2.0181799	1.5	1535	15210	12.2250004	6.2750201
17.75	2.0181799	1.5	1535	15210	12.2250004	6.2750201
17.75	2.0181799	1.5	1535	15210	12.2250004	7.7049799
17.9417	2.08182	1.52	1396	15454	12.8916998	6.60637
18.2000008	1.87273	1.42	1655	14990	12.6917	9.0916796
18.0499992	1.61818	1.41	1787	14577	12.5082998	5.52356
17.8833008	1.76364	1.45	1733	14721	12.2833004	6.7055898
17.6417007	1.70909	1.41	1789	14592	12.2166996	7.0645099
15.3999996	1.54545	1.28	1940	13995	10.625	5.0961499
16.6749992	1.83636	1.36	1812	14520	11.5333004	5.2238402
18.2000008	1.86364	1.4299999	1679	14879	12.5500002	7.7576599
18.0832996	1.93636	1.41	1683	14901	12.6416998	5.8779302
18.8833008	2.0272701	1.47	1484	15353	13.2082996	7.8692298
18.8833008	1.9909101	1.46	1484	15353	13.2082996	6.5952401
18.2000008	1.87273	1.42	1655	14990	12.6917	9.0916796
18.0417004	2	1.85083	1493	15217	12.5749998	5.1856599
18.0333004	1.95455	1.79083	1593	14991	12.7082996	4.6849198
18.0499992	2	1.84417	1506	15231	12.5500002	5.1712799
19.4249992	2.0636401	1.4400001	1551	15368	13.8500004	5.0888901
19.4916992	2.0636401	1.4400001	1548	15390	13.9417	6.1190901
19.4916992	2.0545499	1.4400001	1548	15390	13.9417	6.0124402
19.2833004	2.0636401	1.4400001	1563	15320	13.75	5.45507
19.2166996	2.0090899	1.41	1569	15299	13.6750002	6.7569199
19.4832993	2.0545499	1.4400001	1540	15382	13.8999996	5.0826802
22.8999996	2.09091	1.73	1029	15917	16.8999996	7.4110799
22.7917004	2.09091	1.73	1029	15918	16.8167	6.0471601
22.8999996	2.0999999	1.73	1029	15917	16.8999996	14.4197998
17.7000008	1.93636	1.3200001	1635	15066	12.3832998	6.8295102

18.5667	2.0181799	1.38	1601	15202	13.1167002	8.3836298
18.5	2.0181799	1.37	1614	15176	13.0749998	9.78582
17.2416992	2.0181799	1.3099999	1682	14934	12.0166998	5.9857302
17.2416992	2.0090899	1.3099999	1682	14934	12.0166998	6.60637
17.2416992	2.0090899	1.3099999	1682	14934	12.0166998	6.36515
17.1499996	1.9454499	1.3099999	1695	14865	11.9083004	7.8864999
17.7833004	2	1.37	1677	15030	12.4666996	4.6212201
17.8083	1.9454499	1.35	1666	15012	12.4333	6.7052102
17.9582996	2	1.37	1665	15076	12.6499996	5.98141
18.3083	2.03636	1.39	1636	15166	12.9417	9.3004503
18.5750008	2.03636	1.4	1602	15218	13.1583004	5.8840799
19.4666996	2.0090899	1.83333	1537	15361	13.9417	6.6529102
19.2082996	2.09091	1.8099999	1538	15376	13.6917	6.3281498
19.2583008	2.0181799	1.80667	1554	15340	13.7333002	9.1987305
23.3582993	2.08182	1.7	1034	15874	17.2416992	5.6876798
23.3582993	2.11818	1.73	1034	15874	17.2416992	11.3896999
23.4750004	2.08182	1.73	1007	15913	17.3332996	6.5069399
24.2000008	2.1545501	1.77	906	16045	18.5167007	8.8562698
24.6000004	2.11818	1.77	910	15995	18.7583008	7.6307702
24.3167	2.11818	1.77	904	16031	18.6166992	11.0021
23.0667	2.03636	1.74	1030	15901	17.0916996	9.62140515
22.8167	2.0636401	1.72	1037	15900	16.8167	11.2364998
19.1082993	2.0999999	1.41	1450	15464	13.6999998	8.0063105
24.1000004	2.16364	1.75	940	15987	18.2749996	9.1970901
24.4249992	2.1454501	1.77	912	16001	18.5667	8.3273802
24.0083008	2.12727	1.73	958	15944	18.1082993	6.3590202
24.6749992	2.1818199	1.78	886	16089	19.7999992	8.38908985
24.7000008	2.2	1.78	882	16081	19.7082996	9.2555199
24.7166996	2.2	1.78	884	16063	19.625	7.5226598
23.0832996	2.08182	1.73	1080	15844	17.2082996	7.2799001
23.0832996	2.0727301	1.73	1080	15844	17.2082996	7.1457701
23.2917004	2.08182	1.74	1022	15932	17.2999992	7.1760802
23.2917004	2.0999999	1.74	1013	15931	17.2667007	6.95294
23.3666992	2.0727301	1.74	1011	15908	17.2833004	6.7955298
23.3666992	2.0727301	1.74	1011	15908	17.2833004	6.7955298
24.2000008	2.1545501	1.77	906	16045	18.5167007	8.2158003
24.2000008	2.1545501	1.77	906	16045	18.5167007	6.0342498
24.2000008	2.1454501	1.77	906	16045	18.5167007	6.5321598
24.1667004	2.1818199	1.76	916	16024	18.4582996	6.7055898
24.1667004	2.1818199	1.76	916	16024	18.4582996	6.7706199
24.1667004	2.1545501	1.76	916	16024	18.4582996	8.1631203
24.2000008	2.16364	1.77	906	16045	18.5167007	7.037045
24.0916996	2.1090901	1.75	938	15977	18.2250004	6.2698898
24.5832996	2.11818	1.76	925	15991	18.75	7.8042002
24.1082993	2.17273	1.76	928	15995	18.3167	6.23066
24.4750004	2.1818199	1.77	900	16035	18.7166996	6.31247
23.7166996	2.09091	1.7	1010	15889	17.7583008	10.6886997

Slope (dégré)	Slope (%)	NDVI	Curvature	Aspect_pente	Landcover
2.07726	4.616133333	0.411765	0.0016952	258.0910034	Savane/Marai
7.9037399	17.56386644	0.310345	0.0011992	189.9440002	Zone batie
7.61868	16.9304	0.357143	0.0011839	287.1480103	Mosaique cha
3.84025	8.533888889	0.306452	0.0008366	297.0469971	Mosaique cha
8.6641302	19.25362267	0.333333	0.0010343	14.5004997	Plantation for
6.8980298	15.32895511	0.495146	-0.0003091	47.4550018	Savane/Marai
10.4040003	23.12000067	0.409836	0.0005245	87.3729019	Savane/Marai
2.9546599	6.565910889	0.408451	-0.0000601	251.6660004	Plantation for
5.2528901	11.67308911	0.454545	0.0005208	108.3339996	Savane/Marai
12.5914001	27.98088911	0.369369	0.0005639	356.2349854	Mosaique cha
7.1762099	15.94713311	0.479167	0.0004652	244.8359985	Plantation for
13.5612001	30.13600022	0.394495	0.0000872	343.3829956	Savane/Marai
5.6065502	12.45900044	0.354167	-0.0005323	18.0020008	Savane/Marai
5.0876298	11.305844	0.345794	0.0020851	72.5031967	Mosaique cha
10.2033997	22.67422156	0.319149	0.0000882	93.8562012	Savane/Marai
6.3538399	14.11964422	0.405941	0.0001413	159.3350067	Plantation for
3.6245201	8.054489111	0.396226	-0.0000041	196.0330048	Plantation for
4.6941199	10.43137756	0.378641	-0.0007572	136.3829956	Savane/Marai
2.10304	4.673422222	0.390476	0.0004957	88.0363998	Mosaique cha
4.8730202	10.82893378	0.386139	0.0023182	11.9962997	Savane/Marai
9.8940601	21.98680022	0.344828	0.001439	30.1053009	Mosaique cha
3.23277	7.183933333	0.44186	0.0004953	91.3889008	Savane herbe
3.23277	7.183933333	0.44186	0.0004953	91.3889008	Savane herbe
2.32219	5.160422222	0.450549	0.0002615	82.4499969	Savane herbe
2.32219	5.160422222	0.428571	0.0010283	108.3330002	Savane herbe
5.4031301	12.00695578	0.493333	0.0000564	31.8262005	Savane arbust
6.8285899	15.17464422	0.458333	-0.000389	324.9660034	Savane/Marai
2.10304	4.673422222	0.529412	-0.000107	48.6372986	Savane herbe
1.46917	3.264822222	0.460674	0.0002492	321.1740112	Savane/Marai
10.4040003	23.12000067	0.538462	0.0030299	141.022995	Foret seconda
9.1795797	20.399066	0.5	0.0006508	4.8490601	Savane herbe
0.734703	1.632673333	0.423077	-0.0002147	274.3729858	Savane/Marai
18.5758991	41.27977578	0.295455	0.0011253	274.0360107	Savane/Marai
0.657146	1.460324444	0.381443	0.0007708	273.4830017	Savane/Marai
9.3148804	20.69973422	0.402062	0.001438	295.5109863	Savane/Marai
5.4031301	12.00695578	0.493333	0.0000564	31.8262005	Savane arbust
18.5608006	41.24622356	0.46087	-0.0012604	241.5319977	Plantation for
15.4828997	34.40644378	0.492308	-0.0010212	250.8650055	Plantation for
9.6657896	21.47953244	0.448276	0.0001515	328.0889893	Savane/Marai
10.4782	23.28488889	0.326087	0.0011522	300.2709961	Savane/Marai
7.5211501	16.71366689	0.484536	0.0005504	8.1750498	Savane/Marai
4.2004299	9.334288667	0.408163	0.0014805	244.151001	Savane/Marai
7.30755	16.239	0.206612	0.0011856	180	Mosaique cha
1.99791	4.4398	0.347826	0.000487	185.2870026	Savane/Marai
10.5421	23.42688889	0.297297	-0.0003304	350.0289917	Mosaique cha
1.03897	2.308822222	0.4	-0.0001479	168.6289978	Savane arbust
4.0576	9.016888889	0.456311	0.0001126	307.7210083	Savane/Marai
0	0	0.447154	0.0003353	144.3119965	Plantation for
1.85812	4.129155556	0.469027	0.0000273	134.8399963	Savane/Marai

4.3137202	9.586044889	0.447761	0.0004482	26.6935997	Savane/Marai
7.6875601	17.08346689	0.544828	0.0003531	2.96732	Plantation for
4.3137202	9.586044889	0.367521	-0.0000746	355.8909912	Savane/Marai
2.32219	5.160422222	0.4	0.0017317	198.5319977	Mosaique cha
2.9546599	6.565910889	0.410853	0.001262	207.4819946	Plantation for
5.7934799	12.87439978	0.490566	-0.0005617	77.9718018	Savane herbeu
16.4477997	36.550666	0.403846	-0.0004233	296.4349976	Savane herbeu
16.3771992	36.393776	0.350427	-0.0002395	320.9400024	Mosaique cha
8.6156397	19.145866	0.362069	0.0013781	287.4320068	Mosaique cha
5.0135798	11.14128844	0.338028	-0.0003689	41.1464005	Savane/Marai
9.4811096	21.06913244	0.340206	0.0011408	306.8739929	Savane/Marai
6.6237202	14.71937822	0.495798	178.628006	178.628006	Plantation for
9.1282902	20.28508933	0.294118	225.7160034	225.7160034	Savane/Marai
5.71946	12.70991111	0.465116	135.9019928	135.9019928	Savane arbust
5.80266	12.8948	0.42069	-0.0004324	12.7910995	Plantation for
3.3790901	7.509089111	0.442177	-0.0004197	11.8837996	Savane/Marai
2.56463	5.699177778	0.416058	0.0001471	139.2449951	Mosaique cha
1.46917	3.264822222	0.439394	0.0000559	354.7780151	Savane/Marai
2.50086	5.557466667	0.401575	-0.0001337	203.3070068	Savane/Marai
0	0	0.415385	-0.0001946	314.848999	Savane/Marai
3.915460033	8.096299778	0.475319	-0.0009593	33.5061507	Plantation for
3.28228	7.293955556	0.44	-0.0007164	11.3711004	Savane/Marai
4.0043898	8.898644	0.510638	-0.0012022	55.641201	Savane/Marai
1.91526	4.256133333	0.401361	0.0000072	331.572998	Mosaique cha
2.07726	4.616133333	0.446809	-0.000343	203.3079987	Savane/Marai
2.97281	6.606244444	0.426573	0.0000051	345.3919983	Savane/Marai
1.093352	2.429671111	0.442478	-0.00001865	266.9275055	Plantation for
0.328584	0.730186667	0.469027	-0.000219	185.2200012	Savane/Marai
1.85812	4.129155556	0.415929	0.0001817	348.6350098	Savane/Marai
1.18457	2.632377778	0.464567	-0.0000845	288.3410034	Savane/Marai
2.7075801	6.016844667	0.44186	0.0000584	327.8529968	Savane/Marai
1.3141201	2.920266889	0.438017	0.0003854	76.0374985	Savane/Marai
1.64249	3.649977778	0.477477	0.0001069	211.102005	Savane/Marai
3.84025	8.533888889	0.217391	0.0003006	326.1650085	Mosaique cha
3.84025	8.533888889	0.217391	0.0003006	326.1650085	Mosaique cha
0.929304	2.06512	0.426471	-0.0000025	225.151001	Savane/Marai
4.1101198	9.133599556	0.395683	0.0003567	341.473999	Savane/Marai
2.50086	5.557466667	0.44186	0.0001771	283.3125	Savane/Marai
2.10304	4.673422222	0.411765	0.0001319	213.0429993	Mosaique cha
1.97075	4.379444444	0.449275	0.0003557	321.9779968	Savane/Marai
1.46917	3.264822222	0.504132	-0.0000853	328.9030151	Savane/Marai
2.67620005	5.947111222	0.4038645	0.00010585	340.2810059	Savane/Marai
3.2493601	7.220800222	0.414286	-0.0000602	320.5620117	Mosaique cha
2.10304	4.673422222	0.393443	0.0002719	360	Mosaique cha
3.3790901	7.509089111	0.369863	0.0002394	302.3340149	Mosaique cha
3.1141801	6.920400222	0.464567	0.0004331	324.3200073	Savane/Marai
1.99791	4.4398	0.393103	0.0001885	12.5930996	Mosaique cha

CEC	Density part	WC	Clay	K	Ca	Mg	Na
21	0.7	29	46	1.88	2.47	1.61	0.143
21	0.7	29	46	1.88	2.47	1.61	0.143
21	0.7	29	46	1.88	2.47	1.61	0.143
23	1.4	26	32	0.989	6.71	4.73	2.6199999
35	1.6	31	51	1.61	4.9499998	2.3499999	0.722
36	1.4	30	50	1.74	4.6100001	2	0.305
30	0.9	29	46	1.37	5.1700001	2.0699999	0.163
36	1	32	37	1.9400001	5.0500002	1.74	0.198
29	1.1	30	43	1.5599999	4.3000002	1.6900001	0.184
29	1.1	31	47	1.29	4.5	1.92	0.145
28	1.2	31	49	1.72	4.9899998	2.23	0.306
33	1.3	30	40	1.47	3.5	1.72	0.177
28	1.2	31	49	1.72	4.9899998	2.23	0.306
28	0.9	30	50	1.34	3.76	1.99	0.391
28	0.9	30	49	1.41	4.1799998	2.0899999	0.419
29	1.2	30	49	1.17	3.27	1.77	0.173
18	0.3	27	46	1.5599999	4.2800002	1.8200001	0.0684
16	0.4	27	45	1.47	3.21	1.89	0.0515
29	1.4	29	40	2.04	4.8899999	1.78	0.0814
31	1.4	29	47	1.64	4.6999998	1.54	0.0323
30	1.5	28	40	2.02	5.0900002	1.88	0.0929
29	2.2	29	43	1.96	3.3299999	1.24	0.0793
29	2.2	29	40	1.9400001	4.1100001	1.73	0.0733
33	0.7	30	47	1.38	4.5599999	2.2	0.154
32	1.2	31	47	1.72	2.73	1.67	0.0476
31	0.9	30	45	1.86	2.3599999	1.72	0.0338
30	0.9	30	42	1.79	3.55	1.95	0.0673
31	1.1	31	43	1.6799999	2.22	1.52	0.0478
31	1	32	43	1.74	3.46	2.1099999	0.0874
31	1.1	31	43	1.6799999	2.22	1.52	0.0478
32	1.3	32	45	1.71	2.6300001	1.67	0.129
30	1.8	33	48	1.62	2.23	1.5	0.0531
29	1.5	32	46	1.79	1.77	1.52	0.0466
31	1.1	31	45	1.65	2.02	1.73	0.0529
31	1.1	31	45	1.65	2.02	1.73	0.0529
31	0.9	30	45	1.65	2.3	1.8	0.0384
33	1.4	31	51	1.26	4.5100002	2.0999999	0.15
21	1.4	25	40	1.86	3.5	1.6900001	0.11
33	2.2	30	47	1.37	4.9699998	2.25	0.169
14	1.4	25	36	1.66	2.29	1.28	0.103
17	0.3	23	34	0.815	8.3000002	4.8000002	3.79
21	0.3	23	24	0.501	8.0100002	4.5799999	2.5
20	0.2	25	30	0.989	12.8999996	10.1999998	6.4000001
20	0	24	32	0.697	8.6700001	6.0300002	3.6900001
21	0.1	24	35	0.597	13.3000002	8.7799997	8.2200003
19	0.5	23	30	0.62	8.8400002	4.21	5.4000001
16	0.4	20	28	0.615	8.46	4.8699999	17.7999992
16	0.3	20	28	0.615	8.46	4.8699999	17.7999992
17	0.9	19	21	1.04	6.6500001	4.04	18.5

14	0.1	18	19	0.769	6.8699999	4.04	2.4000001
13	0.2	18	18	0.784	7.5599999	4.0599999	1.85
15	0.2	18	22	0.435	7.96	5.1300001	0.814
16	0.1	17	26	0.47	7.3600001	3.25	1.45
17	0.2	20	25	0.729	7.6300001	4.1799998	0.734
14	0.9	18	22	1.05	8.8599997	4.23	1.27
16	0.2	20	28	0.437	7.23	3.5999999	1.3099999
18	0.2	21	30	0.453	7.5999999	3.5	1.4400001
17	0.1	21	31	0.475	7.54	3.79	1.39
18	0.3	20	27	0.471	7.7800002	4.0799999	2.99
16	0.6	18	23	0.533	7.9400001	4.5	0.606
12	0.2	18	16	0.892	10.3000002	5.1500001	14.8999996
16	0.9	17	27	0.511	8.1400003	3.75	2.54
17	0.3	18	27	0.511	8.0699997	4.04	2.3699999
17	0.1	19	27	0.529	7.8699999	4.3800001	2.77
20	0.2	19	26	0.567	6.8499999	4.6799998	1.5
20	0.9	20	28	0.548	5.5599999	3.3499999	0.668
20	0.2	22	25	0.496	5.2399998	4.46	2.2
21	0.2	23	28	0.385	6.2600002	4.3099999	1.13
19	0.9	20	23	0.402	5.8400002	3.6099999	1.04
18	0.3	22	27	0.769	9.4300003	3.3900001	4.04
19	0.9	22	32	0.785	10.5	3.6700001	5.27
18	0.9	22	31	0.64	11.6000004	3.9300001	4.5300002
17	0.7	26	41	1.9	2.52	1.03	0.0302
20	1.1	26	38	2.02	2.25	1.03	0.0101
26	0.8	30	42	1.46	2.5699999	1.54	0.0231
23	0.9	30	42	1.51	2.6400001	1.47	0.0157
17	1	26	38	2.3199999	2.54	1.33	0.0647
16	0.7	26	34	2.04	2.8800001	1.38	0.156
16	0.7	26	34	2.04	2.8800001	1.38	0.156
24	1.3	28	41	1.61	2.3599999	1.15	0.00244
28	1.1	29	40	1.54	2.0999999	1.26	0.0163
24	1.1	28	42	1.97	2.6600001	1.45	0.00796
14	0.5	24	36	1.8099999	1.75	1.17	0.0208
14	0.6	24	45	1.97	2.1800001	1.36	0.0118
26	0.7	29	42	1.65	3.51	1.6900001	0.017
24	0.6	29	42	1.7	3.7	1.5700001	0.0151
22	0.8	29	41	1.59	3.3499999	1.67	0.0115
30	0.7	28	45	1.6799999	2.3299999	1.59	0.0163
26	0.7	27	40	1.51	2.6700001	1.58	0.0282
20	0.6	26	38	2.22	2.5799999	1.38	0.0202
19	0.9	25	34	0.97	10.3000002	5	4.6500001
14	0.1	21	29	0.824	8.9700003	4.3600001	3.0999999
20	0.1	24	34	0.97	10.3000002	5	4.6500001
15	0.9	26	39	1.52	2.3199999	1.59	0.0864
14	0.7	25	48	1.65	2.05	1.34	0.11
23	0.8	30	46	1.42	2.6800001	1.51	0.1

TSB	N	Field capacity	pH	SAND	Phosphore (ppm)
5	0.227	36	5.2	29	17.49613601
5	0.227	36	5.2	29	12.05564142
5	0.227	36	5.2	29	14.77588872
15	0.872	48	5.5	42	7.666151468
10	0.638	48	5.1	23	7.913446677
9	0.568	46	5	24	7.789799073
8	0.507	41	5.1	31	28.438949
10	0.473	81	5.3	32	22.62751159
9	0.49	44	5	32	25.53323029
9	0.46	40	5.1	26	9.459041731
10	0.675	51	5.1	24	8.28438949
8	0.487	64	5.2	29	8.871715611
10	0.675	51	5.1	24	30.35548686
9	0.442	41	5	25	66.08964451
9	0.436	41	5.1	27	48.22256569
7	0.4	44	5.2	28	12.24111283
7	0.271	31	5.2	36	6.676970634
6	0.291	29	5.2	38	9.459041731
8	1	44	5.1	37	21.14374034
7	1.03	47	5	33	20.71097372
8	0.884	43	5	39	20.92735703
6	0.868	46	5.1	37	27.69706337
8	1.0700001	48	5.1	39	36.16692427
7	0.571	31	5.5	31	31.93199382
5	0.305	56	5	34	36.66151468
5	0.312	50	5	36	48.16074189
7	0.377	52	5	37	42.41112828
5	0.229	58	4.8	34	20.89644513
7	0.323	56	5	33	23.12210201
5	0.229	58	4.8	34	22.00927357
5	0.263	56	4.8	32	25.10046368
5	0.244	56	4.8	29	24.35857805
4	0.293	62	4.8	31	24.72952087
6	0.294	58	5.1	33	27.07882535
6	0.294	58	5.1	33	20.95826893
6	0.308	59	5.1	34	24.01854714
8	0.296	31	5.2	29	35.48686244
5	0.16	33	5.1	42	28.37712519
9	0.38	40	5.3	29	31.93199382
3	0.26	34	5.4	46	36.66151468
20	0.757	27	6.4	43	42.9057187
18	0.682	20	6.2	53	39.78361669
35	1.85	38	6.5	40	46.55332303
22	0.96	24	6.1	47	48.28438949
30	1.3099999	27	6.6	39	47.41885626
24	0.776	25	6.2	48	60.46367852
15	0.859	20	6.7	52	49.95363215
15	0.859	20	6.7	52	55.20865533
13	0.795	17	6.3	59	27.44976816

12	0.639	12	6.1	63	24.48222566
14	0.637	12	6.1	64	25.96599691
13	0.737	12	6.4	57	25.96599691
12	0.628	17	6.3	56	24.42040185
11	0.576	20	6.8	57	25.19319938
14	0.752	14	6.2	54	14.2812983
14	0.711	18	6.2	54	16.38330757
14	0.754	18	6.1	52	15.33230294
14	0.728	18	6.2	50	31.71561051
16	0.662	14	5.9	53	38.02163833
13	0.719	17	6	62	34.86862442
14	1.08	8	5.4	70	13.53941267
14	0.639	17	5.9	56	13.35394127
15	0.639	19	5.8	55	13.44667697
16	0.698	15	5.9	54	38.02163833
12	0.437	16	6	55	43.33848532
9	0.387	17	5.8	52	40.68006182
12	0.558	17	6	50	32.14837713
13	0.543	21	6	46	29.42812983
10	0.526	20	6	54	30.78825348
20	0.764	21	6.5	48	59.84544049
20	1.02	24	6.4	39	58.91808346
25	0.878	19	6.6	43	59.38176198
4	0.377	44	5	39	26.52241113
3	0.274	46	5.1	40	25.03863988
5	0.399	52	5.2	35	25.7805255
5	0.342	44	5.2	37	39.8763524
5	0.377	47	5.1	40	40.68006182
5	0.347	38	5.2	41	40.27820711
5	0.347	38	5.2	41	57.187017
3	0.316	42	5.1	40	56.50695518
3	0.296	34	5.3	40	56.84698609
4	0.344	33	5.2	40	53.60123648
3	0.301	36	5.1	43	49.82998454
4	0.428	45	5	34	51.71561051
4	0.419	37	5.3	38	43.09119011
4	0.407	41	5.1	39	33.19938176
5	0.425	37	5.3	41	38.14528594
4	0.41	25	5.5	37	33.26120556
4	0.431	25	5.5	43	36.16692427
5	0.218	43	5.2	38	34.71406491
22	1.0599999	34	6.4	42	60.21638331
19	0.92	22	6.2	49	61.57650696
22	1.0599999	34	6.4	42	60.89644513
4	0.299	25	5.6	42	31.71561051
4	0.318	49	5.2	31	31.96290572
5	0.249	45	4.9	36	31.83925811

Carbone (%)	MO	C/N	Fréquence%	Intensité%	Sporees desnsity
0.739344262	1.271672131	3.257023182	60.1	15.0	326.7
0.627868852	1.079934426	2.765942081	66.7	20.3	299.3
0.683606557	1.175803279	3.011482632	58.9	19.0	336.7
0.462295082	0.795147541	0.530154911	56.7	11.7	48.0
0.47704918	0.82052459	0.747725988	65.0	15.1	60.3
0.469672131	0.807836066	0.826887555	61.7	14.4	77.3
0.503278689	0.865639344	0.992660135	66.3	10.4	267.3
0.604918033	1.040459016	1.278896475	60.0	11.3	262.0
0.554098361	0.95304918	1.130812981	55.6	9.8	258.7
0.996721311	1.714360656	2.16678546	43.8	11.7	316.7
1.563934426	2.689967213	2.316939891	54.3	11.4	302.0
1.280327869	2.202163934	2.629009998	48.3	10.1	307.7
1.62295082	2.79147541	2.404371585	56.7	11.7	238.0
1.768852459	3.04242623	4.00192864	61.7	14.4	252.3
1.695901639	2.91695082	3.889682659	59.2	13.0	211.0
0.298360656	0.513180328	0.745901639	65.7	18.2	229.3
0.486885246	0.837442623	1.796624524	69.2	22.7	202.7
0.392622951	0.675311475	1.349219762	70.0	25.8	205.3
0.659016393	1.133508197	0.659016393	35.0	10.2	217.3
0.816393443	1.404196721	0.792614993	45.0	10.3	207.3
0.737704918	1.268852459	0.834507826	45.0	11.5	204.3
1.244262295	2.140131148	1.433481907	55.0	10.0	130.0
1.537704918	2.644852459	1.437107266	40.0	9.8	115.0
1.390983607	2.392491803	2.436048348	50.0	12.6	122.3
1.591803279	2.737901639	5.219027143	35.0	13.7	74.7
2.116393443	3.640196721	6.783312316	40.0	10.9	52.7
1.854098361	3.18904918	4.918032787	38.0	14.2	49.3
1.139344262	1.959672131	4.975302455	45.7	9.4	110.0
1.03442623	1.779213115	3.202557986	40.0	10.2	118.0
1.086885246	1.869442623	4.746223781	39.5	9.3	100.7
1.032786885	1.776393443	3.926946332	58.9	9.7	95.3
1.56557377	2.692786885	6.416285945	54.1	10.1	23.7
1.299180328	2.234590164	4.434062552	61.0	11.4	20.7
0.901639344	1.550819672	3.066800491	45.2	10.3	92.3
0.727868852	1.251934426	2.475744396	38.5	11.2	57.7
0.814754098	1.401377049	2.645305514	40.1	9.4	63.3
1.126229508	1.937114754	3.80482942	56.0	11.6	128.7
1.836065574	3.158032787	11.47540984	45.0	10.8	119.3
1.481147541	2.54757377	3.897756687	45.0	11.3	142.0
1.439344262	2.475672131	5.53593947	35.0	13.8	164.0
1.695081967	2.915540984	2.239209996	39.1	15.3	153.3
1.567213115	2.695606557	2.297966444	42.5	11.6	159.3
1.006557377	1.731278689	0.544085069	56.1	18.5	85.3
1.126229508	1.937114754	1.173155738	54.0	15.4	78.3
1.066393443	1.834196721	0.814040858	49.6	23.4	70.3
1.827868852	3.143934426	2.355501099	29.5	9.9	496.0
1.931147541	3.32157377	2.248134506	20.4	10.4	384.0
1.879508197	3.232754098	2.188018855	35.0	9.7	381.7
1.039344262	1.787672131	1.307351273	63.3	13.1	229.3

1.172131148	2.016065574	1.834321045	59.3	12.2	223.0
1.105737705	1.901868852	1.73585197	55.8	12.7	224.0
1.055737705	1.815868852	1.432479925	62.0	21.9	85.7
1.295081967	2.227540984	2.062232432	50.8	15.8	52.3
1.175409836	2.021704918	2.040642077	52.0	18.9	62.7
2.13442623	3.671213115	2.838332752	38.3	11.7	137.7
1.67704918	2.88452459	2.35871896	41.7	10.1	117.7
1.905737705	3.277868852	2.527503587	30.0	10.0	124.0
1.840983607	3.166491803	2.528823635	35.0	9.4	99.7
1.749180328	3.008590164	2.642266356	40.0	11.1	96.3
1.795081967	3.087540984	2.49663695	45.0	10.2	80.7
1.168852459	2.01042623	1.082270795	42.5	10.4	170.0
1.352459016	2.326229508	2.116524282	44.0	11.3	34.7
1.260655738	2.168327869	1.97285718	52.0	12.5	38.7
1.560655738	2.684327869	2.235896472	21.7	7.4	434.0
1.86557377	3.208786885	4.26904753	26.4	6.3	426.7
1.713114754	2.946557377	4.426653111	20.0	6.2	433.3
2.180327869	3.750163934	3.907397614	15.6	5.8	326.7
2.026229508	3.485114754	3.731546056	23.9	5.3	401.0
2.103278689	3.617639344	3.998628685	29.0	5.5	415.0
2.093442623	3.600721311	2.740108145	38.3	5.9	433.3
2.216393443	3.812196721	2.172934748	33.7	5.8	390.0
2.154918033	3.706459016	2.454348557	30.1	5.8	428.3
1.696721311	2.918360656	4.500587033	22.3	6.3	449.3
1.639344262	2.819672131	5.983008257	38.5	6.1	429.7
1.668032787	2.869016393	4.1805333	29.5	6.4	259.0
1.272131148	2.188065574	3.719681718	27.0	6.4	293.3
1.221311475	2.100655738	3.239552985	21.0	7.1	276.7
1.246721311	2.144360656	3.592856805	29.2	5.5	303.3
1.209836066	2.080918033	3.486559267	17.8	6.7	414.7
1.255737705	2.159868852	3.973853497	28.2	6.9	364.7
1.232786885	2.120393443	4.164820558	22.4	7.1	404.3
1.268852459	2.18242623	3.68852459	40.5	7.0	340.7
2.880327869	4.954163934	9.569195578	36.0	7.6	274.3
2.074590164	3.568295082	4.84717328	32.5	5.5	316.3
2.213114754	3.806557377	5.281896788	21.2	6.3	298.0
2.721311475	4.680655738	6.686268981	17.0	5.9	213.3
2.467213115	4.243606557	5.805207329	18.3	5.7	263.3
1.901639344	3.270819672	4.638144742	15.3	6.3	212.0
1.87704918	3.22852459	4.355102507	26.7	5.5	200.7
1.889344262	3.249672131	8.6667168	21.7	5.5	232.0
2.081967213	3.580983607	1.964120198	27.8	6.3	266.0
1.878688525	3.231344262	2.042052744	22.4	6.1	270.0
1.980327869	3.406163934	1.868234015	16.0	6.0	267.0
1.524590164	2.622295082	5.098963759	30.1	6.1	269.3
1.486885246	2.557442623	4.675739767	32.5	6.6	269.7
1.505737705	2.589868852	6.047139377	38.8	6.3	273.3
