# Supplementary figures and images for: Site climate more than soil properties and topography shape the natural arbuscular mycorrhizal symbiosis in maize and spore density within rainfed maize (Zea mays L.) cropland in the eastern DR Congo
Source: PLoS One. 2024 Dec 13;19(12):e0312581. doi: 10.1371/journal.pone.0312581 (PMC11642996; doi:10.1371/journal.pone.0312581)

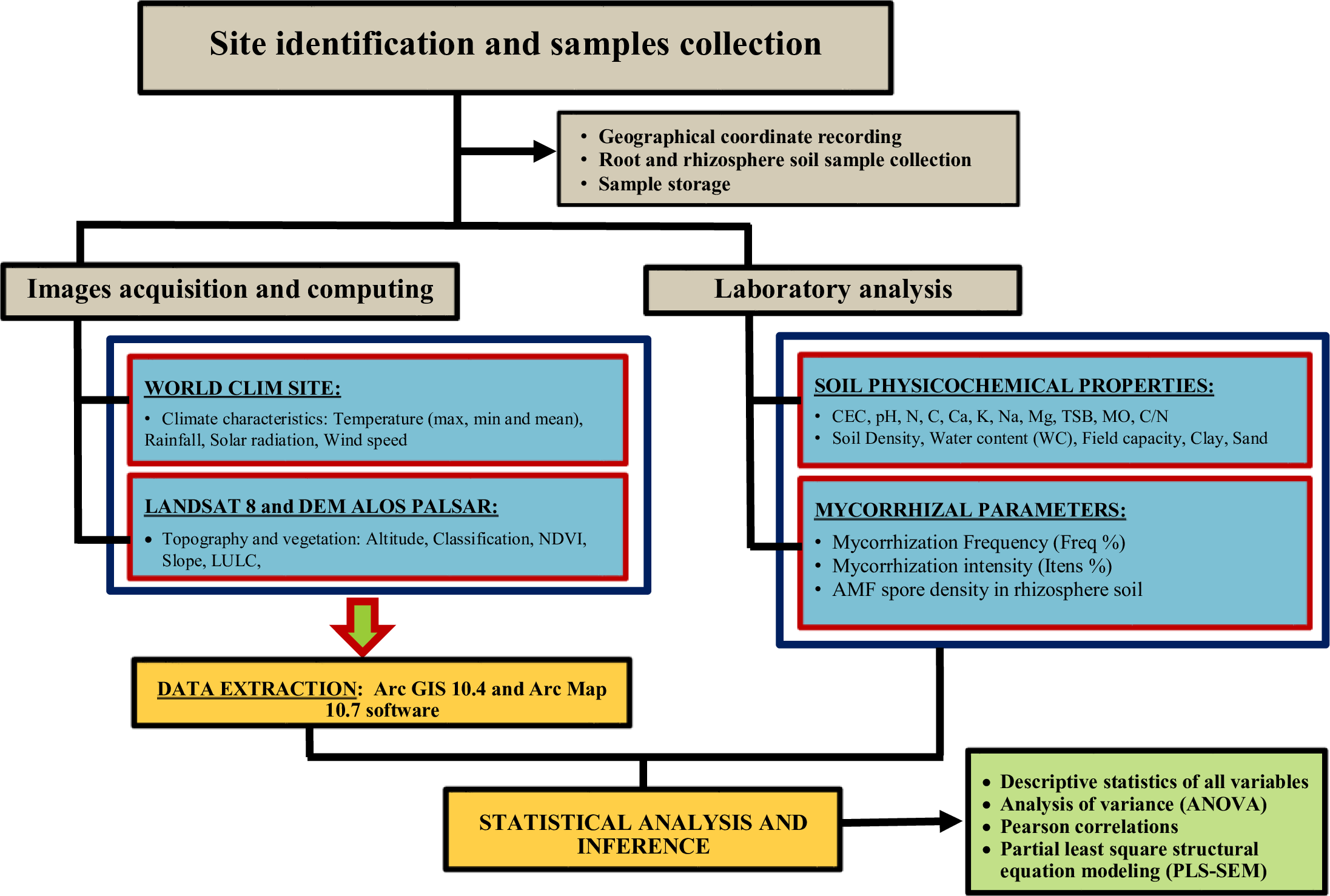

Supplement: S1 Fig — (TIF) [file pone.0312581.s006.tif]

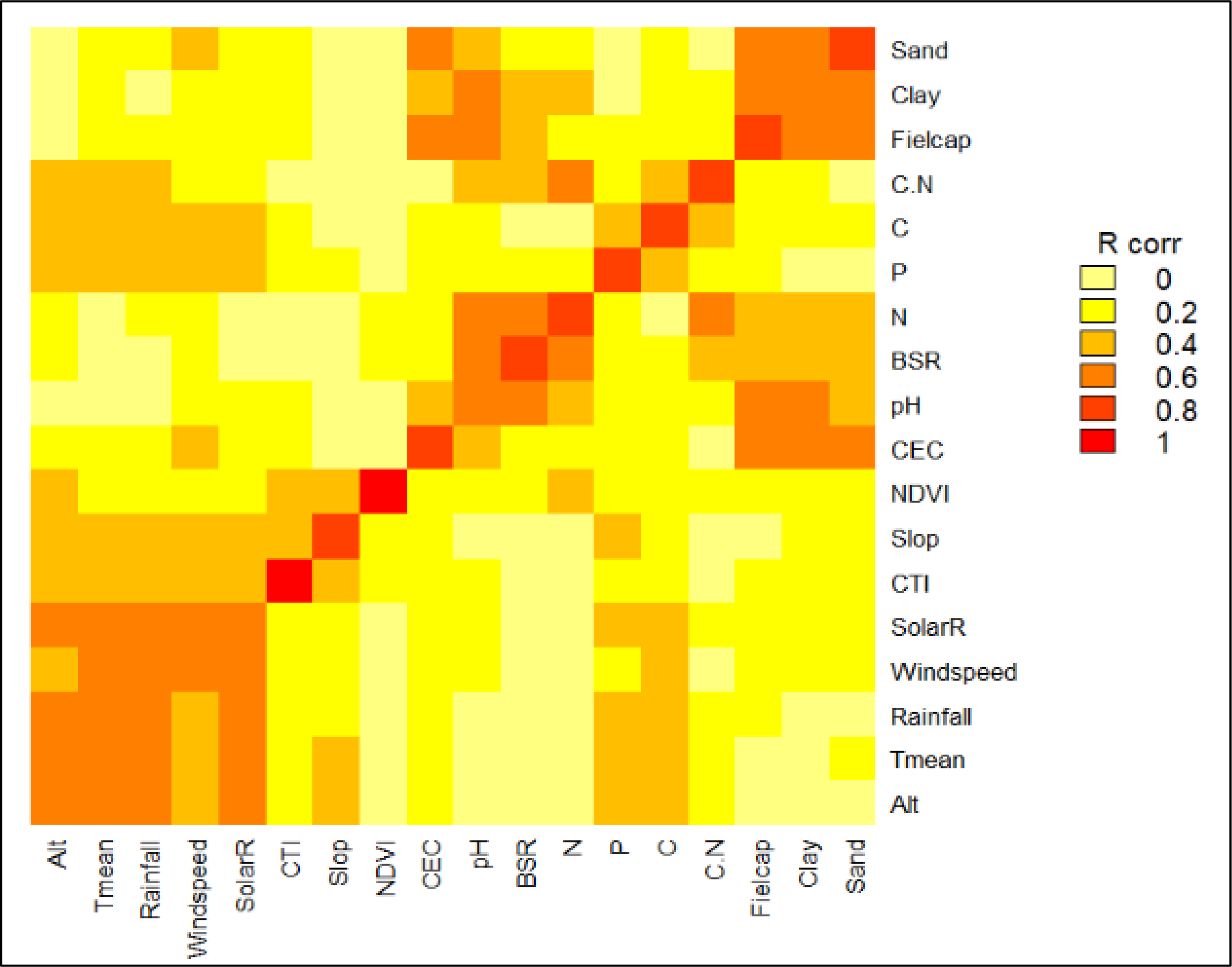

Supplement: S2 Fig — (TIF) [file pone.0312581.s007.tif]

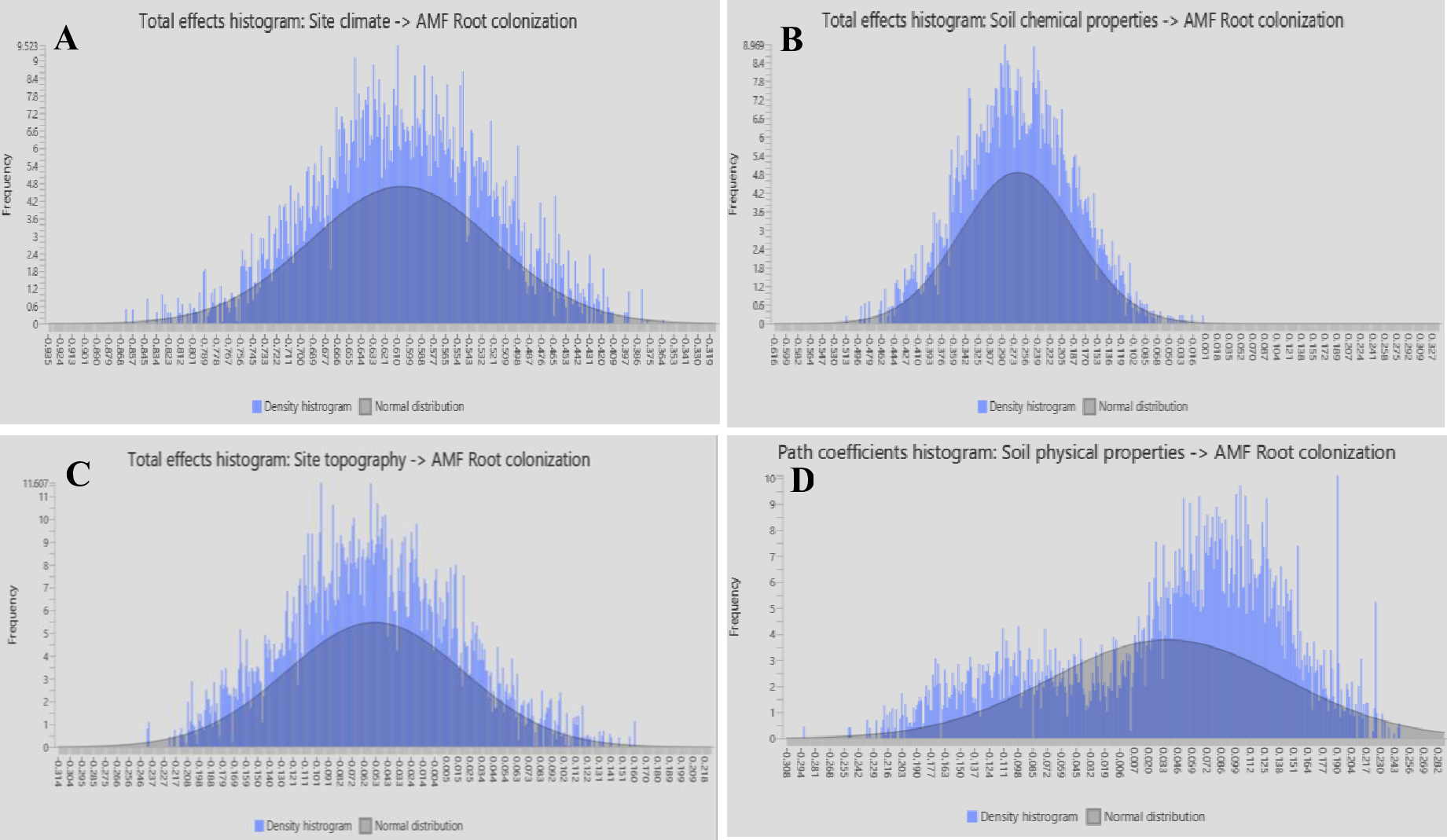

Supplement: S3 Fig — (TIF) [file pone.0312581.s008.tif]

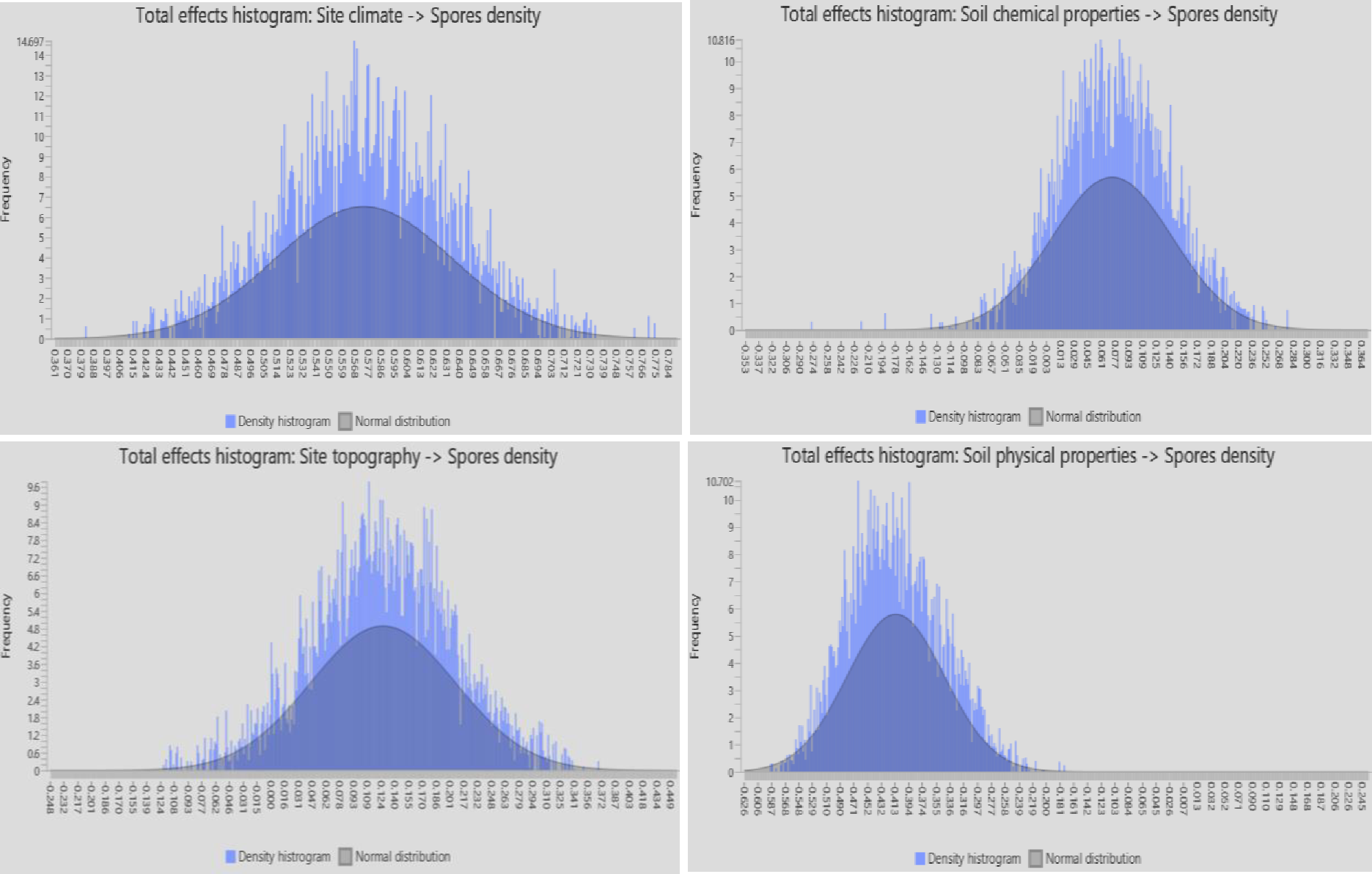

Supplement: S4 Fig — (TIF) [file pone.0312581.s009.tif]
