# Supplementary material for: Site climate more than soil properties and topography shape the natural arbuscular mycorrhizal symbiosis in maize and spore density within rainfed maize (Zea mays L.) cropland in the eastern DR Congo
Source: PLoS One. 2024 Dec 13;19(12):e0312581. doi: 10.1371/journal.pone.0312581 (PMC11642996; doi:10.1371/journal.pone.0312581)
Supplement: Attachment — Submitted filename: Evaluation Report.docx [file pone.0312581.s010.docx]

This study investigated the impacts of site climate, soil properties, and topography on the natural arbuscular mycorrhizal symbiosis in maize and spore density within rainfed maize (*Zea mays* L.) cropland in the Eastern DR Congo. The data were harvested from the 32 sites across three territories. The study employs a well-thought-out experimental design, encompassing site climate variables, soil physicochemical properties, topography, and vegetation variables. This design aids in analyzing the influence of different environmental factors on maize MC and the density of AMF spores and provides reliable data to support the study's conclusions. The article provides a detailed interpretation of the experimental results, highlighting the positive role of site climate factors on arbuscular mycorrhizal fungi colonization and spore density of maize rhizosphere soil. The explanation of the results is clear and well-structured, making it easy for readers to comprehend the study's findings. The authors mention the potential value of the research results in practical field applications. This connection to real-world applications is helpful in translating research findings into actionable recommendations for agricultural production in rainfed maize cropland systems. It is clear that the authors are not concerned about the latest publications in this field in the sections of Introduction and Discussion. The presentations are not sufficient to clarify the key issue and background in terms of AMF colonization. The latest publications might be considered as Rehman et al., 2022, Plant Soil, 1–17; Khan et al., 2024, Science of The Total Environment, 917, 170417, and Abrar et al., 2024, Plant Physiology and Biochemistry, 213, 108839.

In summary, this manuscript has several positive attributes, but minor improvements could further enhance the research.

1) Lines 27: Units of spores density should be uniform.

2) Lines 38: Zea mays should be italicized.

3) Lines 115-117: Reference should be added.

4) Line 118: Replace “bbelow” with “below”.

5) Line 134: A comma should be added after specifically.

6) Line 166: How did plants were uprooted?

7) Lines 168-169: How did rhizosphere soil was collected?

8) Lines 169-170: Arbuscular Mycorrhizal Fungi should be written in lowercase.

9) The equation for the total mycorrhization percentage should be mentioned.

10) Lines 179 and 192: The magnification unit should be standardized.

11) Reference should be added for pH measurement.

12) Reference should be added for exchangeable cations measurement.

13) The instruments and chemicals should be mentioned which were used to determine soil analysis.

14) Line 211: Extra full-stop should be deleted.

15) Lines 38: Full-stop should be deleted after algorithms.

16) In headings 2.7.1, 2.7.2, and 2.7.3, names and versions of software used for analysis should be mentioned.

17) The headings format should be uniform throughout the manuscript.

18) p should be italicized throughout the manuscript.

19) Line 278: Anova should be capitalized and followed throughout the manuscript.

20) Lines 285-287: It should be mentioned that these sites belong to which territory.

21) Line 289: Spore density unit should be corrected (100 g^-1^ soil) and followed throughout the manuscript.

22) Lines 365-366: Arbuscular Mycorrhizal Fungi should be written in lowercase and you have already mentioned the abbreviation earlier, so you have to use an abbreviation instead of using the full name. Follow it throughout the manuscript.

23) Lines 432-435: What are the favorable conditions that promote spore germination?

24) Line 443: Carbon (C) and phosphorus (P) abbreviations should be added at the start and no need to repeat it later.

25) References should be updated by adding journal names, italicizing the scientific names, and keeping the text in a similar pattern such as sentence form.

26) Figure 1 quality should be enhanced.

27) Figure 3, p should be italicized in the figure and caption, Anova should be capitalized, and *p* = < should be written in a standardized form and followed throughout the manuscript.

28) Figure 3, What do highland and lowland stand for? The author did not mention it in the whole manuscript.

29) Table 2, *Zea mays L* should be written as *Zea mays* L.
