# Supplementary material for: Site climate more than soil properties and topography shape the natural arbuscular mycorrhizal symbiosis in maize and spore density within rainfed maize (Zea mays L.) cropland in the eastern DR Congo
Source: PLoS One. 2024 Dec 13;19(12):e0312581. doi: 10.1371/journal.pone.0312581 (PMC11642996; doi:10.1371/journal.pone.0312581)
Supplement: Attachment — Submitted filename: RESPONSES TO REVIEWERS.docx [file pone.0312581.s011.docx]

**RESPONSES TO REVIEWERS**

Dear Editors and Reviewers,

Please find attached the various responses to the comments raised during the review of my manuscript.

Thank you and best regards.

**EDITOR COMMENTS**

--Figure 3: Mycorrhizal Status of maize (Zea mays L) in the three selected territory

--Replace by in the three selected territories or better, use

**Response: The words “selected territory” was replaced by “selected territories”**

**REVIEWER #1:**

1) Lines 27: Units of spores density should be uniform.

**Response : Spore density Unit was uniformed in all the manuscript**

2) Lines 38: Zea mays should be italicized.

**Response: the word “Zea mays” was italicized accordingly**

3) Lines 115-117: Reference should be added.

**Responses : The reference was added accodingly**

4) Line 118: Replace “bbelow” with “below”.

**Response : the word was written correctly**

5) Line 134: A comma should be added after specifically.

**Response : comma was added**

6) Line 166: How did plants were uprooted?

**Response: This is specified in the text; “The maize plants were delicately uprooted to preserve the integrity of the root system. A circle approximately 15 cm in diameter was traced around each plant, and the soil was dug to extract the entire root system”**

7) Lines 168-169: How did rhizosphere soil was collected?

**Responses: Collected roots, along with the surrounding rhizosphere soils (0-20-25 cm), were combined to create three composite samples**

8) Lines 169-170: Arbuscular Mycorrhizal Fungi should be written in lowercase.

**Response : Arbuscular Mycorrhizal Fungi was written accondingly**

9) The equation for the total mycorrhization percentage should be mentioned.

**Responses: The equation was added accordingly**

10) Lines 179 and 192: The magnification unit should be standardized.

**Responses: The magnification unit was standardized**

11) Reference should be added for pH measurement.

**Responses: Reference was added**

12) Reference should be added for exchangeable cations measurement.

**Responses: Reference was added**

13) The instruments and chemicals should be mentioned which were used to determine soil analysis.

**Response : some instrument and chemicals was mentioned in the text**

14) Line 211: Extra full-stop should be deleted.

**Response: Extra full-stop was deleted accondingly**

15) Lines 38: Full-stop should be deleted after algorithms.

**Response: Full-stop was deleted after algorithms accordingly**

16) In headings 2.7.1, 2.7.2, and 2.7.3, names and versions of software used for analysis should be mentioned.

**Response: names and versions of software used for analysis was mentioned in the text**

17) The headings format should be uniform throughout the manuscript.

**Responses : The headings format was uniformized throughout the manuscript**

18) p should be italicized throughout the manuscript.

**Responses : p was italicized in the text**

19) Line 278: Anova should be capitalized and followed throughout the manuscript.

**Responses : Anova was capitalized whirhin the manuscript**

20) Lines 285-287: It should be mentioned that these sites belong to which territory.

**Responses : The origine of each site was specified in the text**

21) Line 289: Spore density unit should be corrected (100 g-1 soil) and followed throughout the manuscript.

**Responses : Spore density unit was corrected (100 g-1 soil) in all the manuscript**

22) Lines 365-366: Arbuscular Mycorrhizal Fungi should be written in lowercase and you have already mentioned the abbreviation earlier, so you have to use an abbreviation instead of using the full name. Follow it throughout the manuscript.

**Responses ; Arbuscular Mycorrhizal Fungi was written in lowercase**

23) Lines 432-435: What are the favorable conditions that promote spore germination?

**Responses : The favourable conditions for spore germination was specified in the text**

24) Line 443: Carbon (C) and phosphorus (P) abbreviations should be added at the start and no need to repeat it later.

**Responses : Carbon (C) and phosphorus (P) abbreviations was written correctly**

25) References should be updated by adding journal names, italicizing the scientific names, and keeping the text in a similar pattern such as sentence form.

**Responses: All references cited was chacked and written correctely in the text and in the reference list**

26) Figure 1 quality should be enhanced.

**Responses : Figure quality was improved. Se in the text (tables and figures file)**

27) Figure 3, p should be italicized in the figure and caption, Anova should be capitalized, and p = < should be written in a standardized form and followed throughout the manuscript.

**Response : This letters was generated with the R software as presented in the main text**

28) Figure 3, What do highland and lowland stand for? The author did not mention it in the whole manuscript.

**Response : highland and lowland stand for agroecological zones**

29) Table 2, Zea mays L should be written as Zea mays L.

**Response : the word was well written as *Zea mays* L.**

**REVIEWER #2:**

**Summary**

- It is well written, but it is important to add the study hypothesis.

Response: The study hypothesis was added in the summary of the manuscript

**Introduction**

- Extremely long, with paragraphs with poorly connected construction;

**Response : The introduction was amended accordingly**

- It is important to add the hypothesis at the end of the introduction text.

**Response :Hypothesis was added at the end of the introduction**

**Methodology**

- Inform whether the Trypan Blue, used to color the roots, was prepared in lactoglycerol or lactophenol;

**Response : Trypan Blue, used to color the roots, was prepared in lactophenol. This were specified in the text**

- Inform the concentration of sucrose used to extract the glomerospores;

**Response : The sucrose concentrations used were specified in the text (two concentrations were used simultaneously during the centrifugation process: 20% sucrose and 60% sucrose).**

- It is important to build a heat map between the variables studied;

**Response : The heatmap between variable was added in the supplementary materialsl**

- Add algorithm analysis (K-means), to check which variables are most relevant in the study;

**Response : We used a multifactorial analysis (multiple factor analysis) that allowed us to group the sites based on their characteristics. We did not apply the K-means method.**

- It is important to add data on the Most Probable Number of AMF infective propagules, production of Soil Proteins Related to Glomalin (PSRG) and spore viability.

**Response : The Most Probable Number (MPN) was not evaluated during the study, nor was the dosage of Proteins Related to Glomalin (PSRG). However, for spore viability, it was specified that only viable spores were counted during the study**

**Discussion**

- Needs adjustments, as there are excessive comparisons with other works. It is important to explain the observed behaviors, especially based on whether or not the hypothesis is confirmed;

**Response : Some adjustments to the discussion have been made and the amendments have been added.**

- There is a need to further explore the effects of C and P on the mycorrhizal parameters evaluated;

**Response : We have amended the aspects regarding the effect of C and P on natural maize mycorrhization and spore density by presenting additional recent arguments.**

- The discussion on sporulation needs to consider what is already well established for fungal reproduction, such as the buffering effect of the substrate on the production of glomerospores.

**Responses : Too few new studies on AMF sporulation are available; however, we were able to add some recent information. See the article by Mukhongo et al., 2023.**

**Conclusion**

- They must be direct and written with the verb in the present tense; are written as a mix of results and final considerations.

**Response : The conclusion was amended accordingly**

**Figures**

- Figures 1 and 7 – improve resolution;

- Figures 2 and 3 – supplementary material.

**Response : Figure 1 and 7 resolution was improved, and Figure 2 was added in the supplementary material**

**Tables**

- Delete table 3;

- Tables 5 and 6 – supplementary material

**Response : Table 3 was deleted and Table 5 and 6 was puted in the supplementary material**

**References**

- It is important to add more up-to-date references on the topic.

**Response : some updated referencies was added in the text**
